# Imaging heterochromatin in human embryonic stem cells with light-sheet Bayesian microscopy

**DOI:** 10.1186/1756-8935-6-S1-P109

**Published:** 2013-04-08

**Authors:** Ying S Hu, Quan Zhu, James Fitzpatrick, Inder M Verma, Hu Cang

**Affiliations:** 1 Waitt Advanced Biophotonics Center, Salk Institute for Biological Studies, La Jolla, CA 92037, USA; 2 Laboratory of Genetics, Salk Institute for Biological Studies, La Jolla, CA 92037, USA

## 

We present a novel super-resolution microscope for the study of chromatin architecture. In eukaryotic cells, chromatin is organized into two distinct domains: lightly packed, and actively transcribed euchromatin, and highly condensed, and transcriptionally silent heterochromatin. While being transcriptionally repressive, heterochromatin is known to serve as binding sites for regulatory proteins, and therefore, plays an important role in the epigenetic regulation of gene expression [1]. However, despite its importance, the architecture of heterochromatin remains elusive, due to the lacking of powerful tools. Single-molecule super-resolution microscopy [2,3], invented by Betzig [4], Hess [5], and Zhuang [6], improves the spatial resolution of light microscopy by over an order of magnitude to ~20-30 nm, and therefore offers a new approach to examine the 3D architecture of chromatin. However, its fundamental requirement of high signal-to-noise ratio (SNR) of fluorescence signal to detect single fluorophore has limited its applications mainly to the study of the cytoskeleton and membrane proteins that reside near the surface of a cell [7-9]. To image beyond the surface of a cell where chromatin resides, we develop a Light-Sheet Bayesian Super-resolution Microscope (LSB-SRM). This microscope is a marriage between two recently developed techniques: light-sheet single molecule super-resolution microscopy and Bayesian super-resolution image reconstruction algorithm. The light-sheet condenser selective illuminates the nucleus of a cell, suppresses the fluorescence background from fluorophore in unwanted layer of the cell, and therefore increases the signal-to-noise ratio of the single-molecule detection. The Bayesian algorithm can efficiently resolve single molecules in images with noisy background. A combination of the two allows us to directly visualize heterochromatic structures in the nucleus of human embryonic stem cells at super-resolution. Our study shows that the heterochromatin organize into nanometer scale domain structures in H1, human embryonic stem cells, which is consistent with the topological domain theory derived from recent sequencing experiments [10,11].

## References

[B1] Maison C Almouzni G HP1 and the dynamics of heterochromatin maintenance Nat Rev Mol Cell Biol 2004 5 296 304 10.1038/nrm1355 15071554

[B2] Patterson G Davidson M Manley S Lippincott-Schwartz J Superresolution imaging using single-molecule localization Annual Review of Physical Chemistry 2010 61 345 367 10.1146/annurev.physchem.012809.103444 20055680 PMC3658623

[B3] Moerner WE New directions in single-molecule imaging and analysis Proceedings of the National Academy of Sciences 2007 104 12596 12602 10.1073/pnas.0610081104 PMC1937512 17664434

[B4] Betzig E Imaging Intracellular Fluorescent Proteins at Nanometer Resolution Science 2006 313 1642 1645 10.1126/science.1127344 16902090

[B5] Hess ST Girirajan TPK Mason MD Ultra-High Resolution Imaging by Fluorescence Photoactivation Localization Microscopy Biophysical Journal 2006 91 4258 4272 10.1529/biophysj.106.091116 16980368 PMC1635685

[B6] Rust MJ Bates M Zhuang XW Sub-diffraction-limit imaging by stochastic optical reconstruction microscopy (STORM) Nature Methods 2006 3 793 795 10.1038/nmeth929 16896339 PMC2700296

[B7] Huang B Wang W Bates M Zhuang X Three-Dimensional Super-Resolution Imaging by Stochastic Optical Reconstruction Microscopy Science 2008 319 810 813 10.1126/science.1153529 18174397 PMC2633023

[B8] Kanchanawong P Nanoscale architecture of integrin-based cell adhesions Nature 2010 468 580 584 10.1038/nature09621 21107430 PMC3046339

[B9] Xu K Babcock HP Zhuang X Dual-objective STORM reveals three-dimensional filament organization in the actin cytoskeleton Nat Methods 2012 9 185 188 10.1038/nmeth.1841 22231642 PMC3304438

[B10] Lieberman-Aiden E Comprehensive Mapping of Long-Range Interactions Reveals Folding Principles of the Human Genome Science 2009 326 289 293 10.1126/science.1181369 19815776 PMC2858594

[B11] Dixon JR Topological domains in mammalian genomes identified by analysis of chromatin interactions Nature 2012 485 376 380 10.1038/nature11082 22495300 PMC3356448

